# Targeted proteomics in urinary extracellular vesicles identifies biomarkers for diagnosis and prognosis of prostate cancer

**DOI:** 10.18632/oncotarget.13634

**Published:** 2016-11-26

**Authors:** Tamara Sequeiros, Marina Rigau, Cristina Chiva, Melania Montes, Iolanda Garcia-Grau, Marta Garcia, Sherley Diaz, Ana Celma, Irene Bijnsdorp, Alex Campos, Primiano Di Mauro, Salvador Borrós, Jaume Reventós, Andreas Doll, Rosanna Paciucci, Michiel Pegtel, Inés de Torres, Eduard Sabidó, Juan Morote, Mireia Olivan

**Affiliations:** ^1^ Group of Biomedical Research in Urology, Vall d’Hebron Research Institute (VHIR) and Universitat Autònoma de Barcelona (UAB), Barcelona, Spain; ^2^ Proteomics Unit, Centre de Regulació Genòmica (CRG), Barcelona, Spain; ^3^ Proteomics Unit, Universitat Pompeu Fabra, Barcelona, Spain; ^4^ Department of Pathology, Vall d'Hebron University Hospital, Barcelona, Spain; ^5^ Department of Urology, Vall d'Hebron University Hospital, Barcelona, Spain; ^6^ Department of Urology, VU University Medical Center, Amsterdam, The Netherlands; ^7^ Department of Pathology, VU University Medical Center, Amsterdam, The Netherlands; ^8^ Sanford-Burnham Medical Research Institute, La Jolla, California, USA; ^9^ Sagetis-Biotech; Grup d'Enginyeria de Materials (GEMAT) Institut Químic de Sarrià, Barcelona, Spain; ^10^ Departement of Basic Science, International University of Catalonia, Barcelona, Spain; ^11^ IDIBELL-Bellvitge Biomedical Research Institute, Barcelona, Spain

**Keywords:** prostate cancer, biomarkers, urine, extracellular vesicles, diagnosis

## Abstract

Rapid and reliable diagnosis of prostate cancer (PCa) is highly desirable as current used methods lack specificity. In addition, identification of PCa biomarkers that can classify patients into high- and low-risk groups for disease progression at early stage will improve treatment decision-making. Here, we describe a set of protein-combination panels in urinary extracellular vesicles (EVs), defined by targeted proteomics and immunoblotting techniques that improve early non-invasive detection and stratification of PCa patients.We report a two-protein combination in urinary EVs that classifies benign and PCa patients (ADSV-TGM4), and a combination of five proteins able to significantly distinguish between high- and low-grade PCa patients (CD63-GLPK5-SPHM-PSA-PAPP). Proteins composing the panels were validated by immunohistochemistry assays in tissue microarrays (TMAs) confirming a strong link between the urinary EVs proteome and alterations in PCa tissues. Moreover, ADSV and TGM4 abundance yielded a high diagnostic potential in tissue and promising TGM4 prognostic power. These results suggest that the proteins identified in urinary EVs distinguishing high- and low grade PCa are a reflection of histological changes that may be a consequence of their functional involvement in PCa development. In conclusion, our study resulted in the identification of protein-combination panels present in urinary EVs that exhibit high sensitivity and specificity for PCa detection and patient stratification. Moreover, our study highlights the potential of targeted proteomic approaches–such as selected reaction monitoring (SRM)–as diagnostic assay for liquid biopsies via urinary EVs to improve diagnosis and prognosis of suspected PCa patients.

## INTRODUCTION

Prostate cancer (PCa) is the most frequently diagnosed cancer and the second leading cause of cancer related death among men in developed countries [1]. Nevertheless, the currently used diagnostic methods for PCa detection are far from ideal. Both, prostate-specific antigen (PSA) serum measurement, and digital rectal examination (DRE) present a low specificity. As a result, a significant rate of unnecessary prostate biopsies (PB) is practiced [2]. There is therefore still a clear need for new biomarkers for a fast and reliable diagnosis of PCa.

Because of the prostate location in the body, in direct contact with the urethra, prostate (cancer) secrete products can be detected in urine. Consequently, urine has been intensively studied as a liquid biopsy source of biomarkers for PCa [3–6]. However, the low protein concentration, the presence of salts and the vast dynamic range of protein expression in urine turn it into a particularly complicated fluid for the discovery of protein-based biomarkers [7].

A rich source for prostate-derived products in urine are extracellular vesicles (EVs) such as exosomes, 50-150 nm sized membrane vesicles that are shed by many mammalian cell types, including malignant cells and formed within the endosomal network and released upon fusion of multi-vesicular bodies with the plasma membrane [8]. In the past decade, EVs have been recognized as potent vehicles of intercellular communication, both in prokaryotes and eukaryotes. This is due to their capacity to transfer proteins, lipids and nucleic acids, thereby influencing various physiological and pathological functions of both, recipient and parent cells [8]. Consequently they have been pointed out as a promising easily accessible biomarker reservoir, as their content (such as proteins, lipids, DNA, and RNA) is thought to reflect the molecular composition of their tissue of origin [9–11]. The potential of using EVs as source of PCa biomarkers as a strategy to overcome the dynamic range challenge in urine has generated considerable interest in the last years. When EVs are purified from the whole urine sample, their protein content is highly enriched while at the same time high-abundance soluble proteins are removed, enhancing the detectability of low-abundance proteins [12, 13]. The analysis of the content of EVs harvested from urine appears to have important advantages such as i) urine collection is a non-invasive procedure, ii) the proteomic and genomic material within EVs is protected from enzyme degradation by the vesicles lipid bilayer [14], and iii) EVs are stable after long-term storage at -80°C, which makes prospective studies feasible [13].

One of the most significant challenges involving the use of EVs for the discovery of new biomarkers is the lack of standardized and reliable isolation methods. The isolation of exosomes from biological fluids is rather complex, due to the presence of protein aggregates and other types of vesicles (such as microvesicles and apoptotic bodies), which are frequently co-isolated with the population of interest [15]. Accordingly, the best procedure for EVs purification remains a discussion topic in the field, with ultracentrifugation being the most frequently used method [16, 17].

Nevertheless, previous reports have indicated urinary EVs to be an excellent source of PCa protein biomarkers. In 2009, Mitchell *et al*. analyzed the proteomic content of urinary EVs from 10 healthy donors and 10 PCa patients who were undergoing hormonal therapy prior to radical radiotherapy. In this cohort, PSA and Prostate-Specific Membrane Antigen (PSMA) were found to be present in almost all of the PCa specimens, but not in the healthy donor specimens [18]. A later study characterized EVs preparations in pooled post-DRE urine samples using a shotgun proteomics procedure identifying around 900 proteins [19, 20]. Since then, several studies have emerged to determine the role of extracellular vesicles in urological malignancies [21–23].

In our study, we used a targeted proteomics approach to specifically quantify a set of 64 proteins in urinary EVs in the context of PCa biomarker validation in a cohort of 107 individual urine-derived human samples. Our study resulted in the identification of a protein biomarker combination present in urinary EVs that exhibits better sensitivity and specificity than the currently available biomarkers used for PCa detection and patient stratification. Therefore, the protein combination reported here improves the detection and management of PCa, avoiding the over-diagnosis and over-treatment associated with the currently used screening methods.

## RESULTS

In this work we used a targeted proteomics approach to specifically quantify a set of 64 proteins in urinary EVs in the context of PCa biomarker validation in a cohort of 107 individual urine-derived human samples. Initially a cohort of 107 urine samples was collected after DRE and EVs were isolate. We then measured protein abundance changes in urinary EVs by SRM, and we built protein-based panels for PCa diagnosis and prognosis in urinary EVs, which we also checked in tissue microarrays analysis.

### EVs isolation from post-DRE urine samples

**Figure 1:**
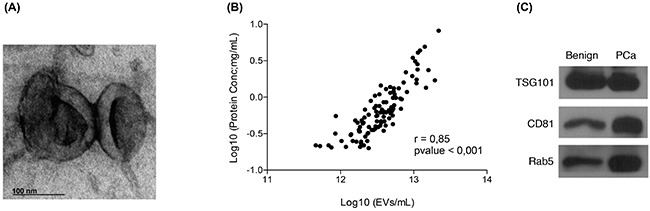
Quality assessment of the EVs isolation process **A**. Transmission electron microscopy images of EVs isolated from post-DRE urine; **B**. Linealregression analysis showing a good correlation between the number of EVs counted by NTA and the total amount of protein recovered from the same sample; **C**. Western blot analysis of TSG101, CD81 and Rab5, described in the literature as EVs markers, were performed in EVs isolated from post-DRE urine. Benign and PCa samples were pooled to obtain representative and sufficient material.

Several approaches were followed in order to assess the purity of the isolated urinary EVs. Urine EVs were observed using a transmission electron microscope (TEM). The TEM images showed vesicles of the expected size, presenting the cup-shaped morphology typical of EVs (Figure 1A) [24]. In addition, NTA and protein quantification data for all samples (n=107) were used to assess the protocol performance. A linear regression analysis showed a good correlation (r=0.85 with p<0.001) between the number of vesicles and total amount of protein extracted, thus indicating that at least an important part of the protein content in the EVs pellet did indeed belong to the vesicles quantified by NTA (Figure 1B). Finally, the presence of known EVs markers, such as TSG101 (tumor susceptibility gene 101), CD81 or Rab5, was confirmed by Western blot (Figure 1C).

### Validation of protein abundance changes in urinary EVs by SRM

The 107 urine samples collected after DRE were divided into two groups: PCa patient samples (n=53), which include 22 low-grade PCa (Gleason score ≤ 7 (3+4)) and 31 high-grade PCa (Gleason score ≥ 7 (4+3)); and control samples (n=54).

A total of 64 protein candidate biomarkers were selected for quantitation by SRM (Supplementary Table ST1). One proteotypic peptide was selected per protein and a scheduled-SRM method was used to allow the monitoring of all 64 proteins in one single run. To ensure that the selected peptides correctly represented the true fold-changes of the targeted proteins, we used a previously in-house shotgun dataset to identify quantotypic peptides for the selected proteins (see Methods section). As a result of these SRM experiments, we observed that 14 out of the 64 initial proteins exhibited a significant different abundance between benign and PCa samples (Table 1), of which 11 had higher protein levels in urine EVs of PCa patients while 3 showed higher levels in urine EVs of control patients. Interestingly, when low- and high-grade PCa samples were compared, 45 out of the 64 measured proteins showed significantly different abundance levels (Table 2), with all proteins exhibiting higher levels in low-grade patients (44 proteins).

**Table 1:** Targeted proteins exhibiting abundance changes in urinary exosomes between PCa patients and benign controls

Gene symbol	Protein	Uniprot accession	FDR	FC (PCa vs. Benign)
*ADSV*	Adseverin	Q9Y6U3	0.002	1.34
*GNS*	N-acetylglucosamine-6-sulfatase	P15586	0.002	1.40
*TGM4*	Transglutaminase-4	P49221	0.002	0.60
*CA4*	Carbonic anhydrase 4	P22748	0.002	1.40
*TSG101*	Tumor susceptibility gene 101 protein	Q99816	0.011	1.22
*VPS28*	Vacuolar protein sorting-associated protein 28 homolog	Q9UK41	0.011	1.20
*SLC44A4*	Choline transporter-like protein 4	Q53GD3	0.013	1.24
*SLC26A4*	Pendrin	O43511	0.020	1.21
*ITGB3*	Integrin β3	P05106	0.020	1.22
*PRSS8*	Prostasin	Q16651	0.020	0.83
*SDCBP2*	Syntenin-2	Q9H190	0.020	1.20
*TSPAN9*	Tetraspanin-9	O75954	0.022	1.17
*ITGAV*	Integrin αV	P06756	0.047	1.20
*PPAP*	Prostatic acid phosphatase	P15309	0.047	0.75

Proteins showing significant protein abundance changes in the SRM analysis (adjusted p-value < 0.05) between benign and PCa patients are shown. Proteins with higher abundance in PCa are represented by fold change values > 1, while values < 1 indicate decreased protein levels in PCa. False discovery rate (FDR); fold change (FC).

**Table 2:** Targeted proteins exhibiting abundance changes in urinary exosomes between high and low-grade patients

Gene symbol	Protein	Uniprot accession	FDR	FC (High vs. Low)
*PPAP*	Prostatic acid phosphatase	P15309	< 0.001	0.37
*PSA*	Prostate-specific antigen	P07288	< 0.001	0.44
*CD63*	CD63 antigen	P08962	< 0.001	0.46
*SPHM*	N-sulphoglucosamine sulphohydrolase	P51688	< 0.001	0.45
*GLPK5*	Putative glycerol kinase 5	Q6ZS86	< 0.001	0.47
*FAM177A1*	Protein FAM177A1	Q8N128	< 0.001	0.46
*GLIPR2*	Golgi-associated plant pathogenesis-related protein 1	Q9H4G4	< 0.001	0.50
*DNASE1*	Deoxyribonuclease-1	P24855	< 0.001	0.54
*STEAP2*	Metalloreductase STEAP2	Q8NFT2	< 0.001	0.58
*TOLLIP*	Toll-interacting protein	Q9H0E2	< 0.001	0.55
*ATP8B1*	Phospholipid-transporting ATPase IC	O43520	< 0.001	0.70
*RRAS*	Ras-related protein R-Ras	P10301	< 0.001	0.67
*CIB1*	Calcium and integrin-binding protein 1	Q99828	< 0.001	0.69
*SLC26A2*	Sulfate transporter	P50443	< 0.001	0.65
*CD82*	CD82 antigen	P27701	< 0.001	0.73
*GALK1*	Galactokinase	P51570	< 0.001	0.69
*TOM1L2*	TOM1-like protein 2	Q6ZVM7	< 0.001	0.64
*ATP6V0D1*	V-type proton ATPase subunit d 1	P61421	< 0.001	0.68
*TMPRSS2*	Transmembrane protease serine 2	O15393	< 0.001	0.65
*PPAP2A*	Lipid phosphate phosphohydrolase 1	O14494	< 0.001	0.57
*GNG4CRYZDBNL*	Drebrin-like protein	Q9UJU6	0.001	0.82
*RPSA*	40S ribosomal protein SA	P08865	0.001	0.74
*GSS*	Glutathione synthetase	P48637	0.001	0.71
*STEAP4*	Metalloreductase STEAP4	Q687×5	0.002	0.68
*PYGL*	Glycogen phosphorylase, liver form	P06737	0.002	0.74
*NUDT2*	Bis(5′-nucleosyl)-tetraphosphatase [asymmetrical]	P50583	0.002	0.65
*TSPAN9*	Tetraspanin-9	O75954	0.003	0.80
*UBC*	Polyubiquitin-C	P0CG48	0.004	0.73
*TMBIM1*	Protein lifeguard 3	Q969×1	0.004	0.70
*FAM49B*	Protein FAM49B	Q9NUQ9	0.005	0.79
*PTPN13*	Tyrosine-protein phosphatase non-receptor type 13	Q12923	0.006	0.78
*LPAR3*	Lysophosphatidic acid receptor 3	Q9UBY5	0.007	0.72
*DPP3*	Dipeptidyl peptidase 3	Q9NY33	0.010	0.73
*GNS*	N-acetylglucosamine-6-sulfatase	P15586	0.013	0.74
*MPI*	Mannose-6-phosphate isomerase	P34949	0.015	0.76
*TSG101DPYS*	Dihydropyrimidinase	Q14117	0.021	0.77
*PCYT2*	Ethanolamine-phosphate cytidylyltransferase	Q99447	0.021	0.80
*VTN*	Vitronectin	P04004	0.024	1.31
*PDCD6IPVPS26AABHD17C*	Alpha/beta hydrolase domain-containing protein 17C	Q6PCB6	0.031	0.87
*ADSV*	Adseverin	Q9Y6U3	0.034	0.81

Proteins showing significant protein abundance changes in the SRM analysis (adjusted p-value < 0.05) between high- and low-grade PCa patients are shown. Proteins with higher abundance in high-grade patients are represented by fold change values > 1, while values < 1 indicate decreased protein levels in high grade patients (i.e. patients with bad prognosis). False discovery rate (FDR); fold change (FC).

**Figure 2:**
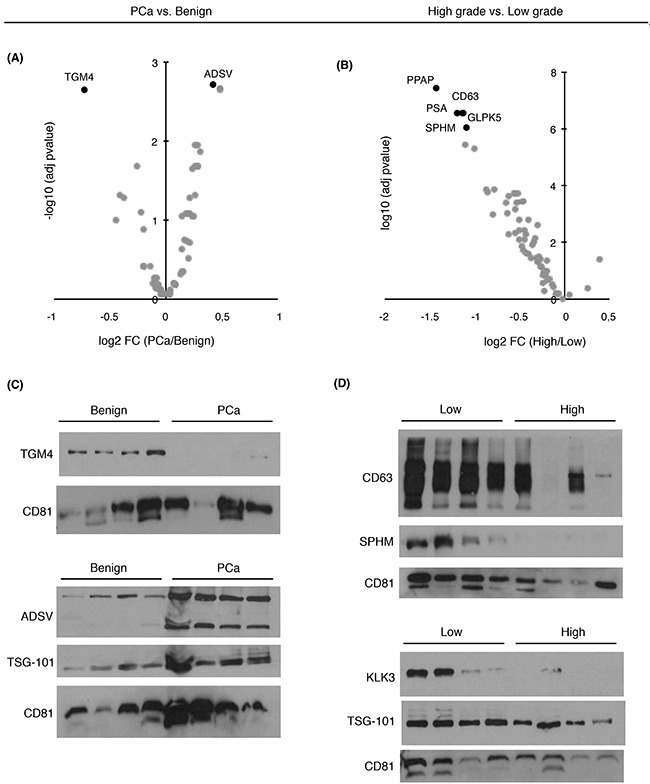
Abundance changes and diagnostic/prognostic evaluation of targeted proteins in urinary EVs Volcano plots represent differentially expressed target proteins in urinary EVs from **A**. PCa vs. benign patients and **B**. from high- vs. low-grade patients; **C**. Western blot of TGM4 and ADSV in a selected set of benign and PCa samples; **D**. Western blot of CD63, SPHM, TSG101 and PSA in a selected set of low and high-grade PCa samples. CD81 was used as EVs related marker.

To evaluate whether the proteins that were significantly changing in abundance as found by SRM-proteomics could be validated by an independent method (Figure 2A-2B), we performed antibody-based immunoblotting in a subset of urinary EVs samples from the same cohort of patients. Among the known EVs markers such as CD9, TSG101 or CD63, protein CD81 was used as an EVs-related marker in our study. Although these are generally accepted by the scientific community as proteins commonly found in EVs [25], they have been found to present different abundance levels in the presence of disease [11]. The results obtained were in agreement with the protein quantitation obtained by SRM (Figure 2C-2D).

### Protein-based panels for PCa diagnosis and prognosis in urinary EVs

**Figure 3:**
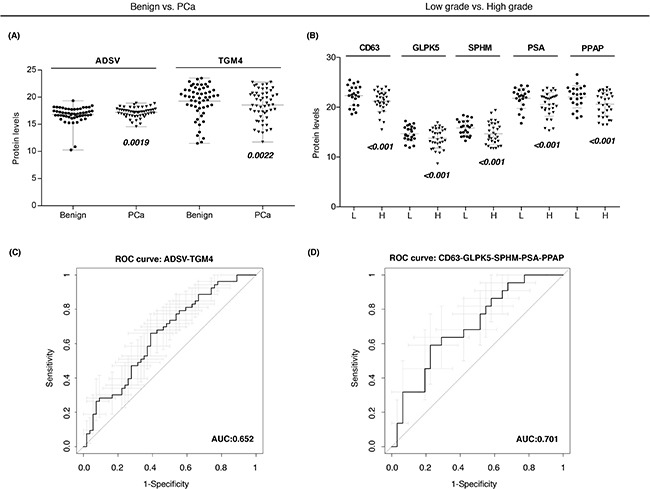
Protein-based panels for PCa diagnosis and prognosis **A-B**. Scatter plots representing the protein abundance levels of selected proteins that are part of diagnostic and prognostic panels, respectively; **C-D**. ROC curves of protein-based panels for PCa diagnosis (ADSV + TGM4) and prognosis (CD63 + GLPK5 + SPHM + PSA + PPAP).

Individual ROC curves and the AUC values were calculated for each individual quantified protein. Previous studies described that combining multiple proteins might improve the diagnostic performance over the use of single biomarkers, as single markers may not necessarily reflect the multifactorial nature of PCa neither have the necessary prediction power for patient classification [26]. Therefore, in order to compute a protein combination with better diagnostic power we fitted abundances of differentially expressed proteins (Figure 3A–3B) into a logistic regression model and checked the AUC for each protein combination. We identified a protein combination formed by TGM4 and ADSV (Figure 3C), which had better sensitivity and specificity (AUC = 0.65, CI95: 0.55-0.76) while the accuracy obtained by each individual protein was low (TGM4 AUC = 0.58; ADSV AUC = 0.58). This result evidenced that the combination of two different proteins improved the ability to distinguish benign from PCa samples.

Next, we also evaluated protein combinations that could correctly classify low and high-grade tumors. Individual markers such as CD63 (AUC = 0.65), GLPK5 (AUC = 0.64), PSA (AUC = 0.66), PPAP (AUC = 0.64) and SPHM (AUC = 0.61) were among the single-protein panels with best sensitivity and specificity. However, it was the combination of these five protein abundances that rendered the best protein panel to distinguish patients with low-grade and high-grade tumors (PPAP + PSA + CD63 + SPHM + GLPK5; AUC = 0.70, CI95: 0.56-0.84) (Figure 3D).

### Tissue microarrays analysis for tissue validation of PCa biomarkers

The most promising EVs biomarkers found in the protein combination models, such as ADSV and TGM4 (for the benign *vs*. PCa comparison) and CD63, GLPK5 and SPHM (for the low-grade *vs*. high-grade comparison) were further evaluated in patient tissues from radical prostatectomies using tissue microarrays (TMAs).

**Figure 4:**
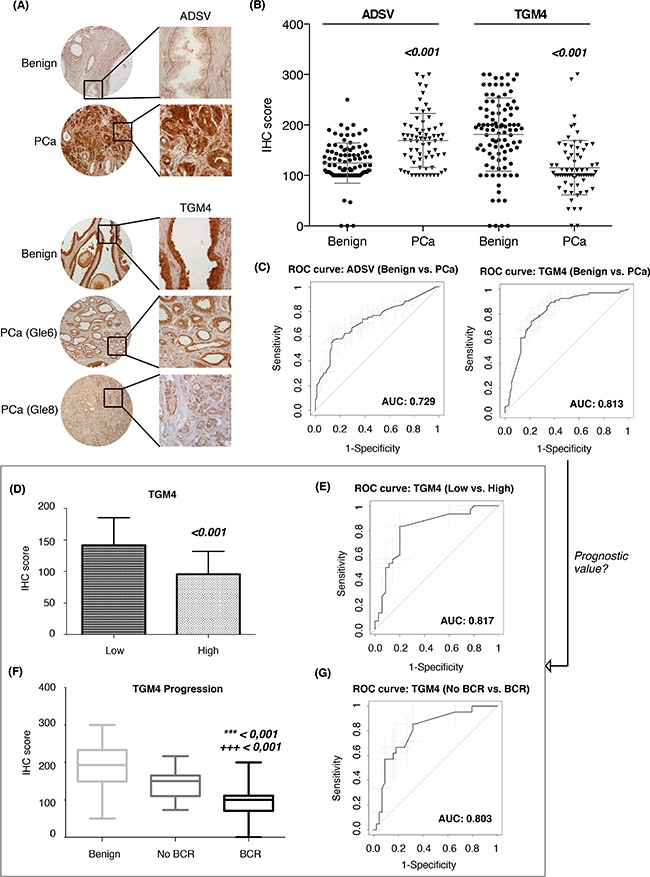
Assessment of PCa diagnostic biomarkers in tissue microarrays **A**. Tissue microarrays images with immunohistochemistry results for ADSV and TGM4; **B**. Scatter plots representing immunohistochemistry scores (IHC) of ADSV and TGM4 in benign and PCa FFPE tissue from PCa patients; **C**. Diagnostic performance represented by a ROC curve of ADSV and TGM4 individually. Prognostic value of TGM4 represented as IHC score **(D)** and ROC curve **(E)** between low vs. high-grade patients. Relation between TGM4 and PCa progression is showed as IHC score **(F)** and ROC curve **(G)** between patients with and without biochemical recurrence (BCR). Values that are significantly different by the Mann-Whitney test from the control group are indicated by p-value < 0.001 (*** vs. benign; +++ vs. No BCR).

In agreement with the expression in urine EVs, IHC on prostate TMA-tissues revealed that both PCa diagnostic biomarkers, ADSV and TGM4, were also altered in terms of protein detection (ADSV p < 0.001; TGM4 p < 0.001) when comparing 98 benign prostatic tissues versus 136 PCa tissues (Figure 4A–4B). We then analyzed the ability of these two proteins to correctly classify patients when directly detected in prostate tissue. ROC curves for both, ADSV and TGM4, individual proteins were generated, obtaining an AUC of 0.81 (CI95: 0.74-0.88) for TGM4, and an AUC of 0.73 (CI95: 0.65-0.81) for ADSV (Figure 4C). Additionally, taking into account the clinical progression of each patient, we tested ADSV and TGM4 as potential prognostic biomarkers and assessed their performance in distinguishing between i) low- versus high-grade patients (as defined for the urinary EVs experiments); ii) groups of patients who presented biochemical recurrence (BCR) versus patients who did not. The analyses revealed that TGM4 could indeed differentiate between the low and high-grade groups with high sensitivity and specificity (p < 0.001; AUC = 0.82, CI95: 0.71-0.92) (Figure 4D–4E) as well as between the BCR versus no BCR groups (p < 0.001; AUC = 0.80, CI95: 0.69-0.91) (Figure 4F–4G). These results suggest that the proteins identified in urinary EVs reflect histological changes and may have a functional role in PCa development since we also find them altered in PCa tissue.

**Figure 5:**
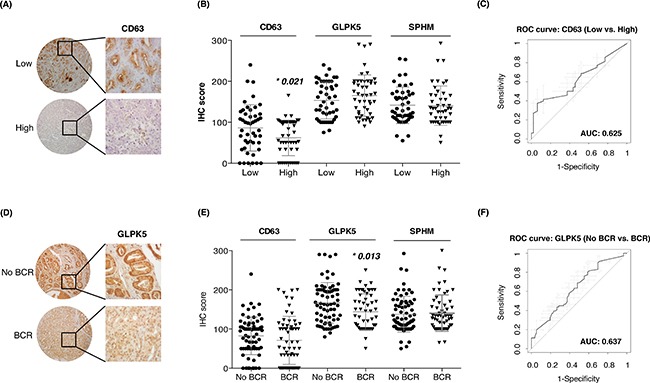
Assessment of aggressive PCa biomarkers in tissue microarrays **A-B**. Scatter plots representing immunohistochemistry scores (IHC) of CD63, GLPK5 and SPHM in PCa FFPE tissue from low and high-grade PCa patients; **C**. ROC curve of CD63 biomarker performance; **D-E**. Scatter plots representing IHC scores of CD63, GLPK5 and SPHM in PCa FFPE tissue from patients with and without biochemical recurrence (BCR); **F**. ROC curve of GLPK5 biomarker performance. Significantly different values were assessed by the Mann-Whitney test and are indicated by *p-value < 0.05.

The profile of urinary EVs proteins obtained by SRM analysis after comparing low versus high-grade PCa patients (CD63, GLPK5 and SPHM), was also validated in tissue samples by using TMAs. CD63 was detected at significantly different levels in tissue between low- versus high-grade PCa patients (p = 0.021), but resulted a modest patient classifier (AUC = 0.62, CI95: 0.51-0.74) (Figure 5A–5C). Similarly, when classifying patients with BCR versus patients without BCR, GLPK5 was able to differentiate between the two groups (p = 0.013; AUC = 0.64, CI95: 0.54-0.73) (Figure 5D–5F) and, furthermore, taking into account only the patients of Gleason 7, the patients that had been developed BCR showed significantly lower levels of GLPK5 (p = 0.0015) (Supplementary Figure SF1). Finally, SPHM did not show, neither significant value when comparing low- versus high-grade PCa patients, nor when comparing PCa patients with and without BCR.

## DISCUSSION

Nowadays, there is still a clear clinical need to identify new biomarkers that improve the early non-invasive detection and stratification of PCa patients. Within this context, the use of EVs as source of new biomarkers is under intense investigation, especially because they can be obtained by non-invasive methods using urine samples.

Recently, transcriptomic analysis of urinary EVs from PCa patients has been carried out with promising results for Cadherin 3, which shows a decreased abundance in PCa samples as a source of biomarkers [27] and the use of microRNAs as markers for this disease has also been extensively reported [28]. In this work, we have performed targeted proteomics and immunoblotting techniques to address this question. Our results show that alterations detected in urinary EVs reflect the protein changes in the prostate tissue and highlight the potential of SRM targeted proteomics as diagnostic assay for liquid biopsies via urinary EVs to improve diagnosis and detect PCa patients with a poor prognosis.

### PCa diagnosis and prognosis protein-based panels in urinary EVs

Current diagnostic techniques for PCa are based on a measurement of serum PSA and DRE, but decisive PCa diagnosis is based on PB, which is indicated when patients present serum PSA above 4 ng/mL. Furthermore, repeat PB is indicated for patients who have a prior negative biopsy but continue to have an elevated serum PSA or abnormal DRE, or as follow-up of previous pathologic diagnoses of pre-malignant HGPIN [29]. The lack of specificity of the above-mentioned tools urges for the identification of new biomarkers using non-invasive methods that improve the early diagnosis of PCa. Although different PSA isoforms have been analyzed across multiple studies; the AUC value of total serum PSA (biomarker used in the clinic) has been estimated to be around 0.6 [30].

In our study, a total of 64 previously identified candidate biomarkers for PCa in urinary EVs were validated in a large cohort of samples (n=107) by SRM. Whereas immunological methods like TMA or ELISA represent the traditional way of validation, targeted MS-based approaches like SRM are emerging as additional alternatives [31]. In this study, SRM methodology was applied in order to facilitate the simultaneous quantification of a large number of candidate proteins in a large cohort of samples. The presence of several of these proteins had already been described in prostate-related urinary EVs by others [19], which proved to be good candidates as PCa biomarkers.

In terms of diagnosis, our urinary EVs diagnostic panel (AUC=0.65) slightly improves the diagnostic performance of serum PSA. However, considering the promising potential that EVs have shown as a source of biomarkers, further studies will be conducted to verify if this diagnostic panel, in combination with current screening tools, could increase the efficiency in detecting PCa and avoid unnecessary biopsies.

From a prognosis point of view, controversy exists regarding the potential of PSA as a prognostic biomarker. Although often serum PSA has higher expression in more aggressive PCa patients [32], it is often found decreased together with several EVs markers, including PSMA, CD9, TSG101, miR-21 and miR-375 among others. In agreement with these observations, we detected decreased PSA levels in urinary EVs [18, 33]. The abundance of PSA within urinary EVs in combination with the protein abundance of four more proteins (CD63, GLPK5, SPHM and PAPP) results in an AUC value of 0.70, for detecting PCa patients with a poor prognosis. These findings are in agreement with a previous report describing that a combination of PSA to CD63 or CD9 improves the detection of PCa. Whether the decrease in prostate-related markers is due to a decrease in the quantity of prostate secreted EVs number within urine, or a change in protein expression profile within prostate (cancer) secreted EVs remains to be clarified [34].

### Changes in EVs protein abundances reflect alterations in prostate tissue

TMAs of PCa patients were used to directly detect each selected protein profile and determine if the observed protein content in urinary EVs reflected changes in the prostate tissue. All patients included in the study presented > 7 years of follow up after surgery, therefore, we were able to correlate the results not only with the presence of the disease and Gleason score but also with their clinical progression (BCR after RP).

The protein abundance changes observed in urinary EVs of ADSV and TGM4 were validated in prostate tissue by IHC analysis and resulted to have even a better AUC value. This result suggest that developing new techniques to pre-selecting certain subtypes of vesicles may lead to better diagnosis performance. ADSV, also known as scinderin (SCIN), has been implicated in the translocation of secretory vesicles directed to be exocytosed [35, 36]. ADSV has been found highly expressed in human PCa tissue, and has been described to be critical for the proliferation of PCa cells [37].

In addition, the IHC analysis also identified a relationship between TGM4 protein levels and Gleason score as well as BCR development decreasing significantly in those patients with a poor prognosis. TGM4 is a protein almost uniquely expressed in the prostate gland [38] and it has been described to be down-regulated in PCa tissue [39]. This protein was previously measured in urinary secretions from patients with extra-capsular or organ-confined PCa. Although its diagnostic power was not evaluated, TGM4 was shown to be down-regulated in poor prognostic PCa [40]. Despite the controversy about the expression of TGM4 in PCa, these previous studies combined with our findings may indicate a possible role of TGM4 as a tumor suppressor gene and, after a further validation in a larger cohort of patients, its potential use not only as a PCa diagnostic biomarker in urinary EVs, but also to identify the poor prognosis patients using biopsies or tissue samples from radical prostatectomy. Thus, ADSV and TGM4 appear to be promising candidates to be confirmed in future studies aimed to establish their possible roles in PCa clinical outcome.

The defined prognostic urinary EVs protein profile (CD63, GLPK5 and SPHM) was also validated by TMA. PSA and PPAP were discarded from the TMA study due to extensive previous research already available in the literature. PSA is strongly expressed in the prostate, both in benign and neoplastic tissue. However, IHC staining detected a lower abundance in cancer compared to the adjacent benign epithelium, which decreased according to less differentiate PCa [41–43]. Similarly, PPAP immunoreactivity has also been demonstrated to be more intense in the benign prostate epithelium and detected at lower levels in PCa [41]. These observations are consistent with the levels we have detected for these two proteins in the urinary EVs. Among the prognostic proteins, it is worth mentioning that CD63 is mainly associated with membranes of intracellular vesicles, although cell surface expression may also be induced. It has been shown that prostate basal epithelial cells do not express the characteristic CD antigens of secretory cells, however the expression levels of CD63 found in cancer cells are similar to that of secretory cells [44]. Surprisingly, our study revealed that decreased protein levels of CD63 in EVs fractions as well as in prostate tissue could indicate poor prognosis of PCa. Similar results were obtained with GLPK5 (glycerol Kinase 5) taking into account the BCR development, however, it is worth noting that this protein was able to distinguish patients with Gleason score 7 who developed BCR from those who did not, therefore, this candidate could be useful in predicting tumor behavior in patients with uncertain risk of progressing. From a functional point of view, further studies will be required to understand the role of this protein in PCa development and progression.

### Urinary EVs as a source of disease biomarkers in liquid biopsies

PCa is a clinically heterogeneous and often multifocal disease with a clinical outcome difficult to predict. Proteomics data give a different level of understanding and together with the innovative high-throughput technologies is a promising way to identify new biomarkers for PCa detection, prognosis and therapy [45]. In this work we combined this strategy with the isolation of urinary EVs to identify new protein biomarker panels for PCa diagnosis (TGM4 and ADSV) and for patient stratification (PSA, PPAP, CD63, GLPK5 and SPHM).

The strategy presented in this manuscript highlights the potential of liquid biopsies via urinary EVs isolation for the diagnosis and PCa stratification, and its associated clinical research. Biomarker detection from urinary EVs is capable of distinguishing aggressive from clinically insignificant PCa and other benign conditions beyond serum PSA and, thus, it might avoid PCa related over-diagnosis and over-treatment. Taking all together, we conclude that the future of the diagnosis and prognosis in PCa will benefit from research of biomarkers in EVs, leading to an improvement in therapeutic decision making with liquid biopsies.

## METHODS

### Experimental design and statistical rationale

This is a retrospective case-control study to identify biomarkers associated to PCa. The main objectives are to assess the potential of urinary EVs as a source of PCa biomarkers and to evaluate a different biomarker candidates previously identified in a larger cohort of patients. The validation cohort was integrated by a total of 107 urine samples obtained after DRE (Table 3). For statistical analysis, PCa cases were divided into low grade PCa (Gleason score ≤ 7(3+4); n=22) and high grade PCa (Gleason score ≥ 7(4+3); n=31). The control group included patients without PCa (n=54), such as patients with benign prostatic hyperplasia (BPH), inflammation or high-grade prostatic intraepithelial neoplasia (HGPIN).

**Table 3:** Clinico-pathological conditions of patients included in the study

	SRM experiments (urine)		TMAs Validation (FFPE)	
	Benign	PCa	Benign	PCa
No. of samples	54	53	98	136
Age (yr)*	65.6 (53 - 78)	67.7 (51 - 87)	64 (53-72)	64 (53-73)
Serum PSA (ng/mL)*	8.1 (2.5 - 41.1)	17.3 (1.0 - 245.4)	9,8 (2,5-48)	11 (1,1-66)
No. of vesicles/mL*	5,1*10^12^ (5,3*10^11^- 2,2*10^13^)	4,1*10^12^ (4,6*10^11^- 1,4*10^13^)	-	-
Total protein (μg/mL)*	1.1 (0.2 - 8.0)	0.8 (0.2 - 4.9)	-	-
Low Grade (GS ≤ 7(3+4))	-	22	51	50
High Grade (GS ≥ 7(4+3)	-	31	48	86
No BCR	-	-	49	72
BCR	-	-	49	64

* Values are represented as mean (range); GS (Gleason's Score)

### Patients selection and inclusion criteria

This study obtained approval from the Vall Hebron University Hospital (Barcelona, Spain) institutional review board (PR(IR)56/2014). Written informed consent was obtained from all the study participants and samples were coded to ensure sample tracking and confidentiality on patient/donor identity. All patients were men with suspicion of PCa according to abnormal DRE and/or serum PSA levels higher than 4 ng/mL, referred for a first PB at the Urology Service of the Vall d’Hebron University Hospital (Barcelona, Spain) from 2008 to 2012. Definitive diagnosis was achieved after PB. Patients with a diagnosis of PCa and age matched controls were selected. Patients with an unrelated chronic or acute severe illness and/or previous PCa therapies were excluded from the study.

### Urine samples collection and extracellular vesicles isolation

Urine was collected after DRE, a procedure included in the standard process for detection of PCa. Urine (30-50 mL) was collected in urine collection cups, kept on ice, transported to the lab and processed within 2 h of its collection. The samples were centrifuged at 2,500 *g* for 10 min at 4°C and the supernatant, containing the EVs, was supplemented with a cocktail of protease inhibitors (Sigma-Aldrich, St Louis, MO, USA) and stored at −80°C until its use.

For EVs isolation, cell-free urine samples were first centrifuged at 16,500 *g*, 20 min, to remove larger vesicles and any possible remaining cell debris. The pellet from this centrifugation was treated with dithiothreitol (DTT) (37°C, 10 min) in order to break THP (Tamm-Horsfall protein) fibers and release EVs, and centrifuged again at 16,500 *g*, 20 min. Supernatants from the two centrifugations were mixed together and filtered through a 0.2 μM pore size filter. Samples were then ultracentrifuged at 100,000 *g* for 120 min at 4°C. The resulting pellet was washed with PBS (phosphate buffered saline) and centrifuged again at 100,000 *g*, 60 min at 4°C. The final pellet was resuspended in 50 μL PBS, of which 5 μL were set aside and stored at -80°C for nanoparticle tracking analysis (NTA) and the rest was mixed with 50 μL lysis buffer (Tris 20 mM pH 8.8, NaCl 150 mM, EDTA 5 mM, Triton X-100 1%, and protease inhibitors) and stored at -20°C until its use.

### Transmission electron microscopy

Transmission electron microscopy (TEM) imaging of EVs was performed using negative-staining technique. Briefly, pellets recovered from the ultracentrifugation, containing the EVs, were fixed with 4% paraformaldehyde and then deposited on Formvar/Carbon-coated grids, which were negatively stained with uranyl acetate. EVs preparations were examined using a transmission electron microscope JEOL JEM 1010 (Japan Electron Optics Laboratory Co., Tokyo, Japan).

### Nanoparticle tracking analysis

Vesicles present in purified samples were analyzed by NTA using the NanoSight LM14 system (NanoSight Ltd., Amesbury, UK), configured with a high sensitivity digital camera system (Hamamatsu C11440 ORCA-Flash2.8, Hamamatsu City, Japan). Three videos of 60s duration were recorded for each sample and average measurements and standard deviations were calculated. Videos were analyzed using the NTA-software (version 2.3), with the minimal expected particle size, minimum track length, and blur set to automatic. Camera shutter speed and camera gain were set to maximum. Camera sensitivity and detection threshold were set close to maximum (15 or 16) and minimum (2 to 5), respectively, to reveal small particles. Samples were diluted in ultrapure water.

### Sample preparation for mass spectrometry

Urinary EVs were disrupted by sonication (LABSONIC M, Sartorius Stedim Biotech, Goettingen, Germany) at 100 % amplitude for 4 pulses of 5 s each separated by 5 s pauses on ice. The extracted proteins were stored at −20°C. An aliquot of each preparation was used for protein quantity estimation using the DC Protein Assay (Bio-Rad, Bio-Rad Laboratories, Hercules, CA, USA), following the manufacturer's instructions. Samples, usually diluted 1:10, were compared in triplicates against serially diluted Bovine Serum Albumin (BSA) as standard.

Filter-aided sample preparation (FASP) was performed using a 10 kDa molecular weight cut-off filter (Merck Millipore) as previously described [46]. Briefly, 20 μg of sample in RIPA buffer were loaded in the filter unit and washed twice with 8 M urea by centrifugation at 14,000 *g* for 15 min. Proteins were reduced with 10 mM DTT for 1 h at RT, and alkylated with 30 mM iodoacetamide (IAA) 30 min in the dark. The reaction was stopped with 37.5 mM n-acetylcysteine (NAC) during 15 min, and the solutions were removed by centrifugation at 14,000 *g* for 15 min. The samples were then washed once with 1 M urea. The resulting pellet was diluted with 40 μL of 1 M urea, containing 20 μg of trypsin, and it was incubated overnight 37°C for protein digestion. Finally, tryptic peptides were collected in a clean tube by centrifugation at 14,000 *g* for 10 min, and this filtrate was acidified with 0.3-0.5 μL of concentrated formic acid. Samples were stored at −20°C until further analysis.

### Selected reaction monitoring

A total of 64 proteins were selected for the targeted proteomic analysis based on previous in-house discovery proteomics experiments and additional information retrieved from the literature. Literature search was focused on previous studies in which differentially expressed proteins in PCa had been identified using tissue, urine and different PCa cell lines as a source of biomarkers [11, 19, 21, 23].

One quantotypic peptide per protein was selected based on a previously in-house shotgun dataset. Briefly, to ensure that the selected peptides correctly represented the true fold-changes of the targeted proteins, we extracted all ion intensity chromatograms from MS1 scans for the identified peptide precursors of the selected proteins across 24 samples. All the ions were visually inspected and area integration of all the precursor ions was manually reviewed. The integrated areas were used to statistically assess protein fold-changes. Peptides were selected for the SRM experiment according the following criteria: i) the difference between the protein and the peptide fold-change (the lower the better); and ii) the intensity of the peptide (the more intense the better). In the case of proteins that exhibited not significant abundance changes, peptides selection was based on peptide intensity.

All samples were individually digested (see above) and one isotopically labeled reference peptide at C-terminal lysine (^13^C_6_,^15^N_2_-Lys) or arginine (^13^C_6_,^15^N_4_-Arg) per protein was spiked into the digested samples prior mass spectrometry (MS) acquisition (Supplementary Table ST1).

Selected reaction monitoring (SRM) measurements were performed with unfractionated samples on a hybrid triple quadrupole / ion trap mass spectrometer (5500 Q-Trap; AB Sciex Instruments, Foster, CA, USA) equipped a reversed-phase chromatography 25-cm column with an inner diameter of 75 μm, packed with 1.9-μm C18 particles (Nikkyo Technos, Tokyo, Japan) and a 2-cm pre-column (Acclaim PepMap 100, C18, 15 μm, 100-A; Dionex, Sunnyvale, CA, USA). Loading buffer: H_2_O + 0.1% formic acid; Elution buffer: ACN + 0.1% formic acid. Flow rate: 250 nL/min. Gradient: From 7 to 40% eluting buffer in 60 min. Blank runs were performed between the SRM measurements of biological samples to avoid sample carryover. Measurements were done in scheduled SRM mode, using a SRM detection window of 300 seconds and a total cycle time of 2.5 seconds. For each peptide of interest five transitions were monitored for both the endogenous and the isotopically labeled peptides.

Transition groups corresponding to the targeted peptides were evaluated with Skyline v2.5 based on the correlation of transition intensities between the endogenous and reference SRM traces as well as with the reference spectral library, on the relative retention times across runs, and on the co-elution of endogenous peptide and spiked-in references [47]. Transition areas were used to calculate protein ratios between the two groups using the linear mixed-effects model as implemented in the R package MSstats v2.0 [48].

The raw proteomics data have been deposited to the ProteomeXchange Consortium [49] via the PASSEL [50] partner repository with the dataset identifier PASS00843.

### Western blotting

Proteins were separated by 10% SDS-PAGE under reducing or non-reducing conditions and transferred to PVDF membranes. For blocking, membranes were soaked in 5% non-fat dried milk in TBS-Tween20 (Tris-buffered saline, 0.01% Tween20). Proteins were immunodetected using antibodies against TSG101 (1:500; Abcam, Cambridge, UK), CD81 (1:100; Santa Cruz Biotechnology, Santa Cruz, CA, USA), CD63 (non-reduced conditions, 1:1000, Merck Millipore, Germany), PSA/KLK3 (1:100, Dako, Denmark), TGM4, ADSV, SPHM (1:1000, 1:100, 1:100, Sigma-Aldrich, MO, USA), in overnight incubations at 4°C. Afterwards, membranes were washed and incubated with a secondary HRP-coupled antibody (horseradish peroxidase-conjugated antibody) for 1h at room temperature. Finally, HRP signal was revealed using the Immobilon Western HRP Substrate (Merck Millipore, Darmstadt, Germany).

### Tissue microarrays

For the tissue microarrays (TMAs) a total of 165 samples of radical prostatectomy (RP) were selected from patients with > 7 years of clinical follow up. Low and high-grade patients and patients with and without biochemical recurrence (BCR) were included. BCR is defined as the first post-operative PSA value > 0.4 ng/mL, confirmed by at least 1 subsequent increasing value (persistent PSA increase) after undetectable PSA post-operatively. From each PCa patient, both cancerous and benign peripheral tissues were inspected. Formalin-fixed paraffin-embedded tissue (FFPE) samples of PCa available from the archives of the Pathology Department of the Vall Hebron University Hospital (Barcelona, Spain) were used. Clinico-pathologic features of these patients are detailed in Table 1. For TMA construction, a hematoxylin and eosin-stained section was made from each block to define representative tumor regions. Tissue cylinders with a diameter of 1 mm were then punched from selected tumor and non-malignant areas of each donor tissue block in triplicates and brought into a recipient paraffin block using a custom-made precision instrument (Advanced Tissue Arrayer Chemicon International USA). Sections (3 μm) of the resulting TMA block were obtained with a Leica RM 2255 microtome (Finesse ME+ A77500016 microtome, Thermo Scientific) and transferred to glass slides and stained with different antibodies as described below.

### Immunohistochemistry

Slides were incubated at 55°C overnight, then treated in xylene to de-paraffinize them, and subsequently re-hydrated through graded alcohol rinses. TGM4, ADSV, GLPK5, CD63 and SPHM immunohistochemical (IHC) staining were performed. Heat-induced antigen retrieval was performed by immersing the slides in 10 mM citrate buffer (C_8_H_8_O_7_.H_2_Odd 0.01M + C_6_H_5_O_7_.H2Odd; pH 6.0). Nonspecific staining was avoided using 10 % normal-matched serum and 0.3 % H_2_O_2_ in PBS buffer for 30 min. Antigen was detected by 1 h incubation at room temperature or overnight at 4°C with the relevant primary antibody (ADSV 1:1000, TGM4 1:1000, GLPK5 1:50, SPHM 1:600, Sigma-Aldrich, MO, USA; CD63 1:500, Merck Millipore, Germany), followed by an appropriate secondary antibody conjugated to a peroxidase complex (Envision+ poly-HRP system; DAKO Cytomation, Glostrup, Denmark). Color development was done using DAB+ Chromogen (DAKO Cytomation) followed by counterstaining with hematoxylin. Two-experienced pathologist evaluated the staining of the TMA and calculated a histoscore based on the percentage of stained cells and the intensity of the staining (ranging from 1 -weakly positive- to 3 -strongly positive). Histoscores were calculated from the sum of (1x % cells staining weakly positive) + (2x % cells staining moderately positive) + (3x % cells staining strongly positive) to a maximum of 300.

### Statistical analysis of mass spectrometric data

SRM peak areas were normalized based on the areas corresponding to the isotopically labeled internal peptide references, which were used to first equalize the median abundance (log2-areas) for all reference peptides across all runs, and then all endogenous peptide areas in a run were corrected by a same bias. Comparisons of relative protein abundance between groups were performed using the MSstats R package [48].

For predictive analysis, the whole patient cohort was used. Initially, the quantity of each protein per sample was estimated based on a relative log2-transformed using the MSstats R package, and these protein quantities were then used as input variables to a logistic regression model between the tested patient groups. Each protein was fitted in the model between two groups and its classification ability was evaluated by the area under the curve (AUC) obtained in the receiver operating characteristic (ROC) curve. AUC was used as it summarizes well the classification performance of each protein in terms of specificity and sensitivity, with candidates with better specificity and sensibility having an AUC value close to 1. Additional proteins were iteratively added and the variation of AUC was checked and only those that increased the AUC value were kept for further iterations [51]. The pROC package in R was used to draw ROCs, calculate the AUC values and estimate the confidence intervals of the ROC curves. Mann-Whitney test and the pROC package mentioned above were used for the IHC data analysis.

## SUPPLEMENTARY FIGURE AND TABLE




