# Supplementary material for: Targeted proteomics in urinary extracellular vesicles identifies biomarkers for diagnosis and prognosis of prostate cancer
Source: Oncotarget. 2016 Nov 26;8(3):4960–76. doi: 10.18632/oncotarget.13634 (PMC5354884; doi:10.18632/oncotarget.13634)
Supplement: Supplementary file 1 [file oncotarget-08-4960-s001.pdf]

Targeted proteomics in urinary extracellular vesicles identifies biomarkers for diagnosis and prognosis of prostate cancer

SUPPLEMENTARY FIGURE AND TABLE

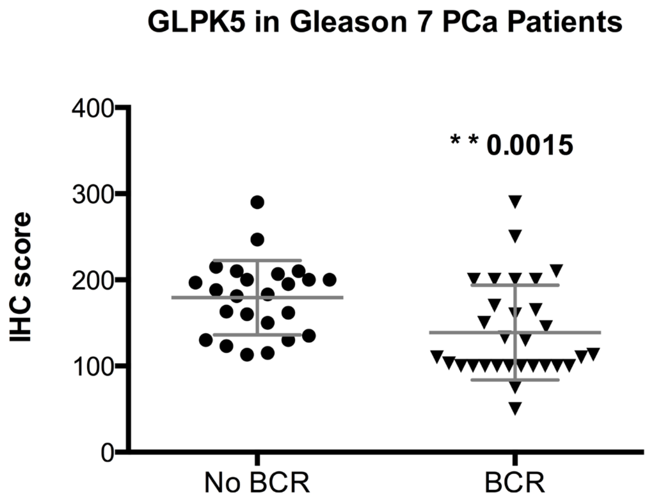

Supplementary Figure S1: Assessment of GLPK5 abundance in tissue microarrays of Gleason 7 PCa patients. Scatter plot representing immunohistochemistry scores (IHC) of GLPK5 in PCa FFPE tissue from patients of Gleason 7 with and without biochemical recurrence (BCR). Significantly different values were assessed by the Mann-Whitney test and are indicated by **p-value < 0.01.

Supplementary table S1: List of the proteins and its corresponding peptide surrogate monitored in the SRM study

See Supplementary File 1
